# Noninvasive respiratory support outside the intensive care unit for acute respiratory failure related to coronavirus-19 disease: a systematic review and meta-analysis

**DOI:** 10.1186/s13054-021-03697-0

**Published:** 2021-07-30

**Authors:** Gianmaria Cammarota, Teresa Esposito, Danila Azzolina, Roberto Cosentini, Francesco Menzella, Stefano Aliberti, Andrea Coppadoro, Giacomo Bellani, Giuseppe Foti, Giacomo Grasselli, Maurizio Cecconi, Antonio Pesenti, Michele Vitacca, Tom Lawton, V. Marco Ranieri, Sandro Luigi Di Domenico, Onofrio Resta, Antonio Gidaro, Antonella Potalivo, Giuseppe Nardi, Claudia Brusasco, Simonetta Tesoro, Paolo Navalesi, Rosanna Vaschetto, Edoardo De Robertis

**Affiliations:** 1 Department of Medicine and Surgery, University of Perugia, Piazza Università 1, 06123 Perugia, Italy; 2 Department of Translational Medicine, University of Eastern Piedmont, Novara, Italy; 3 Emergency Department, ASST Papa Giovanni XXIII, Bergamo, Italy; 4 Pneumology Unit, Arcispedale Santa Maria Nuova, Azienda USL-IRCCS Di Reggio Emilia, Reggio Emilia, Italy; 5 Respiratory Unit and Cystic Fibrosis Adult Center, Fondazione IRCCS Ca’ Granda Ospedale Maggiore Policlinico, Milan, Italy; 6 Department of Pathophysiology and Transplantation, University of Milan, Milan, Italy; 7 ASST Monza, San Gerardo Hospital, Monza, Italy; 8 Department of Medicine and Surgery, University of Milan-Bicocca, Monza, Italy; 9 Department of Anesthesia, Intensive Care and Emergency, Fondazione IRCCS Ca’ Granda Ospedale Maggiore Policlinico, Milan, Italy; 10 Department of Anesthesia and Intensive Care Medicine, Humanitas Clinical and Research Center – IRCCS, Rozzano, Italy; 11 Department of Biomedical Sciences, Humanitas University, Milan, Italy; 12 Respiratory Rehabilitation Unit Lumezzane, ICS Maugeri IRCCS, Brescia, Italy; 13 Department of Anesthesia and Critical Care, Bradford Teaching Hospitals NHS Foundation Trust, Bradford, UK; 14 Anesthesia and Intensive Care Medicine, Policlinico Di Sant’Orsola, Alma Mater Studiorum University of Bologna, Bologna, Italy; 15 Department of Emergency Medicine, ASST Grande Ospedale Metropolitano Niguarda, Milan, Italy; 16 Cardiothoracic Department, Respiratory Unit, University Hospital, Bari, Italy; 17 Department of Biomedical and Clinical Sciences Luigi Sacco, University of Milan, Ospedale Luigi Sacco, Milan, Italy; 18 Department of Anesthesia and Intensive Care, Infermi Hospital, AUSL Della Romagna, Rimini, Italy; 19 Anesthesia and Intensive Care Unit, E.O. Ospedali Galliera, Genoa, Italy; 20 Department of Medicine-DIMED, Università Di Padova, Padua, Italy

**Keywords:** Noninvasive ventilation, COVID-19, Intra-hospital mortality

## Abstract

**Background:**

Noninvasive respiratory support (NIRS) has been diffusely employed outside the intensive care unit (ICU) to face the high request of ventilatory support due to the massive influx of patients with acute respiratory failure (ARF) caused by coronavirus-19 disease (COVID-19). We sought to summarize the evidence on clinically relevant outcomes in COVID-19 patients supported by NIV outside the ICU.

**Methods:**

We searched PUBMED®, EMBASE®, and the Cochrane Controlled Clinical trials register, along with medRxiv and bioRxiv repositories for pre-prints, for observational studies and randomized controlled trials, from inception to the end of February 2021. Two authors independently selected the investigations according to the following criteria: (1) observational study or randomized clinical trials enrolling ≥ 50 hospitalized patients undergoing NIRS outside the ICU, (2) laboratory-confirmed COVID-19, and (3) at least the intra-hospital mortality reported. Preferred Reporting Items for Systematic reviews and Meta-analysis guidelines were followed. Data extraction was independently performed by two authors to assess: investigation features, demographics and clinical characteristics, treatments employed, NIRS regulations, and clinical outcomes. Methodological index for nonrandomized studies tool was applied to determine the quality of the enrolled studies. The primary outcome was to assess the overall intra-hospital mortality of patients under NIRS outside the ICU. The secondary outcomes included the proportions intra-hospital mortalities of patients who underwent invasive mechanical ventilation following NIRS failure and of those with ‘do-not-intubate’ (DNI) orders.

**Results:**

Seventeen investigations (14 peer-reviewed and 3 pre-prints) were included with a low risk of bias and a high heterogeneity, for a total of 3377 patients. The overall intra-hospital mortality of patients receiving NIRS outside the ICU was 36% [30–41%]. 26% [21–30%] of the patients failed NIRS and required intubation, with an intra-hospital mortality rising to 45% [36–54%]. 23% [15–32%] of the patients received DNI orders with an intra-hospital mortality of 72% [65–78%]. Oxygenation on admission was the main source of between-study heterogeneity.

**Conclusions:**

During COVID-19 outbreak, delivering NIRS outside the ICU revealed as a feasible strategy to cope with the massive demand of ventilatory assistance.

**Registration:**

PROSPERO, https://www.crd.york.ac.uk/prospero/, CRD42020224788, December 11, 2020.

**Supplementary Information:**

The online version contains supplementary material available at 10.1186/s13054-021-03697-0.

## Background

The rapid and massive spread of severe acute respiratory syndrome related to novel coronavirus (SARS-CoV-2) outbreak has put in crisis the healthcare systems of whole nations. Worldwide, the surge capacities of the hospitals have been severely stressed by the massive influx of patients admitted for acute respiratory failure (ARF) caused by coronavirus-19 disease (COVID-19) [1–3]. Among COVID-19 patients suffering from hypoxemic ARF, the rate of intubation has been reported ranging from 12 to 33% [3–5]. To face this exceptional demand of intensive care unit (ICU) resources, hospitals have increased ICU bays [6] and adapted many general wards into intermediate care units, with the aim of providing respiratory support and clinical monitoring to those hypoxemic ARF patients in whom the sole conventional oxygen supplement is ineffective [7].

Moreover, at the very beginning of the pandemic, the rate of patients receiving noninvasive respiratory support (NIRS) upon ICU admission was reported to range from 11 [8] (in Italy) to 56% (in China) [9]. At the same time, several studies demonstrated that NIRS outside the ICU was feasible and effective in preventing invasive mechanical ventilation (IMV) [10, 11]. However, a major concern while treating hypoxemic ARF patients by NIRS is related to the failure rate of NIRS, which could occur even in 50% of the cases with consequent recourse to IMV [12]. Also, excessive prolongation of NIRS may worsen lung injury because of patient self-inflicted lung injury occurrence [13] or delay IMV [14, 15].

The aim of this systematic review and meta-analysis was to estimate the overall intra-hospital mortality of COVID-19 patients assisted through NIRS outside the ICU and quantify the proportion of patients who failed NIRS and were subsequently intubated and treated in the ICU. Also, the estimate of patients who received NIRS as a ceiling ventilatory therapy and the related intra-hospital mortality were investigated.

## Methods

Our systematic review and meta-analysis was realized following the Preferred Reporting Items for Systematic reviews and Meta-analysis (PRISMA) guidelines [16] and was registered on PROSPERO (CRD42020224788).

### PICO question

We sought information about the application of NIRS—i.e., continuous positive airway pressure (CPAP) or noninvasive bi-level ventilation—outside the ICU (I) in adult patients admitted for hypoxemic ARF COVID-19 related (P) with or without comparator (C) and aimed to ascertain the intra-hospital mortality (O). For overall intra-hospital mortality we intended the punctual intra-hospital mortality reported by each enrolled study at database closure.

### Search methods and study selection

We searched PUBMED®, EMBASE®, and the Cochrane Controlled Clinical trials register from inception to February 2021 for observational studies and randomized controlled trials without language restrictions. The search was performed using the following terms, combined according to database syntax (see Additional file 1 for search strategy): ‘COVID-19,’ ‘novel coronavirus 2019,’ ‘SARS-CoV-2,’ ‘severe acute respiratory syndrome coronavirus related,’ ‘SARS-CoV-19,’ ‘positive pressure respiration,’ ‘NIV,’ ‘noninvasive ventilation,’ ‘CPAP,’ ‘continuous positive airway pressure,’ ‘noninvasive positive pressure respiration,’ ‘NIPPV,’ ‘NRS,’ and ‘noninvasive respiratory support.’ For NIRS outside the ICU, we meant all the modalities of noninvasive bi-level and CPAP, regardless of the interface used, adopted to assist COVID-19 patients with hypoxemic ARF, with the exceptions of the high-flow nasal cannula.

We also reviewed the references of selected papers, review articles, commentaries, and editorials on this topic to identify other studies of interest missed during the primary search. Moreover, we surveyed medRxiv and bioRxiv, free online repositories for preprints in health science, from inception to end of February 2021, searching for clinical and preclinical investigations about NIRS application in COVID-19 patients outside the ICU.

Two authors (GC and TE) independently evaluated titles and abstracts obtained from the search to select investigations responding to the following inclusion requests: (1) observational study or randomized clinical trials enrolling ≥ 50 symptomatic hospitalized patients undergoing NIRS outside the ICU, (2) laboratory-confirmed COVID-19 defined by a positive result on a reverse-transcriptase-polymerase chain reaction assay of a nasopharyngeal and oropharyngeal swab or a sputum specimen, and (3) at least the primary outcome reported by the study. Case reports and case series with less than 50 patients were excluded, as they may observe no events due to the small size [17]. When multiple publications of the same research group/center described potentially overlapping cohorts, the authors selected the most recent publications. The same authors independently screened the full texts, and any disagreement was resolved through discussion or involving a third review author (EDR). When necessary, the corresponding authors of the included studies were contacted to obtain missing data related to study demographics, methods, outcomes, and clinical characteristics of patients analyzed.

### Data extraction and study quality

Data extraction was independently performed by two authors (GC and TE) who screened and selected the included studies extracted. Any disagreement was resolved by discussion or involving a third review author (EDR). Extracted data included: investigation features (e.g., study design, setting), demographic characteristics (e.g., age, sex, body mass index), presence of comorbidities (with special attention to hypertension, diabetes, kidney disease, respiratory disease, and cardiac disease), characteristics at hospital admission (e.g., oxygenation, respiratory rate, laboratory tests), treatments, NIRS regulations, and clinical outcomes.

The methodological quality of selected articles was assessed by an index that classifies nonrandomized studies as adequate, inadequate, or unclear [18].

### Statistical analysis

The analysis was carried out on the data extracted from peer-reviewed manuscripts in combination with data obtained from pre-print investigations.

The descriptive analysis was conducted for all the selected variables considered in the included studies. Continuous or noncontinuous variables were reported as appropriate. Proportions with 95% confidence intervals (CI) and model fitting weights were computed using the DerSimonian-Laird method with a random-effects model, based on the expected heterogeneity. Heterogeneity across the studies was assessed through Q and I^2^ tests both, which were considered significant when the *p*-value was < 0.05 and *I*^2^ > 75% [19], and graphical evaluation of forest plots.

A general linear (mixed-effects) meta-regression model was performed by using the outcome as the dependent variable and the study size as the independent variable. Meta-regression was conducted to assess, in patients admitted for COVID-19 undergoing NIRS, the impact of age, gender, arterial oxygen tension to inspired oxygen fraction ratio (PaO_2_/FiO_2_) acquired on hospital admission, number of intubations, and number of ‘do-not-intubate’ (DNI) orders patients on the clinical outcomes investigated. Again, the observations were weighted by the inverse variance of the estimate to allow for possible heteroscedasticity.

Statistical analyses were conducted using R3.5.2 software (The R foundation).

## Results

As depicted in Additional file 2, a total of 1956 records were identified from the search, including 1045 peer-reviewed studies and 911 pre-prints studies. After duplicates exclusion and full-text evaluation, 17 eligible studies were identified (14 peer-reviewed and 3 pre-prints) for a total of 18,204 patients with a suspected COVID-19-related infection at hospital admission of whom 3377 received NIRS outside ICU [10, 11, 20–34].

### Characteristics of the included studies

The main characteristics of the included studies are reported in Additional file 3-Table 1. Except for one study conducted in Russia and two investigations performed in the UK, the leading part of the studies was conducted in Italy (82.4%) during the first wave of COVID-19 pandemic, from the end of February to the end of May 2020. Among the 17 enrolled investigations, 11 (64.7%) were single-center studies, whereas 6 (35.3%) were multicenter studies: of these, 2 investigations (33.3%) were prospectively conducted, while 4 were retrospectively carried out. The overall risk of bias was low for the studies included. The methodological quality of the included investigations assessed through methodological index for nonrandomized studies (MINORS) tool is reported in Additional file 3-Table 2 and Additional file 4.

### Patient characteristics

The demographic characteristics are described in Additional file 3-Table 3. A total of 3377 patients were under NIRS outside the ICU. Of these, 2696 (79.8%) were males with an average age ranging from 60 to 75 years and an average body mass index ranging from 27 to 31.9 kg/cm^2^ (2413/3377 patients). The mean Charlson comorbidity index varied from a minimum of 1 to a maximum of 4 (1037/3377 patients). Among comorbidities, hypertension was reported in a higher number of studies compared to other comorbidities. Patients’ clinical characteristics on hospital admission are presented in Additional file 3-Table 4. PaO_2_/FiO_2_ at hospital admission was the most reported clinical variable in the included studies. Additional file 3-Table 5 describes the pharmacological therapies administered and the application of awake-prone position. The rate of hydroxychloroquine administration was the most reported pharmacological therapy among the included investigations. Eight studies reported the application of awake-prone position during NIRS. NIRS settings are described in Additional file 3-Table 6. When reported, CPAP was applied in 2764/3047 of the patients and helmet interface was used in 1855/2690 of the cases. Positive end-expiratory pressure varied from a mean value of 7 to 15 cm H_2_O (2870/3377 patients) and FiO_2_ ranged from a mean value of 50 to 68% (2467/3377 patients), respectively.

### Clinical outcomes

**Fig. 1:**
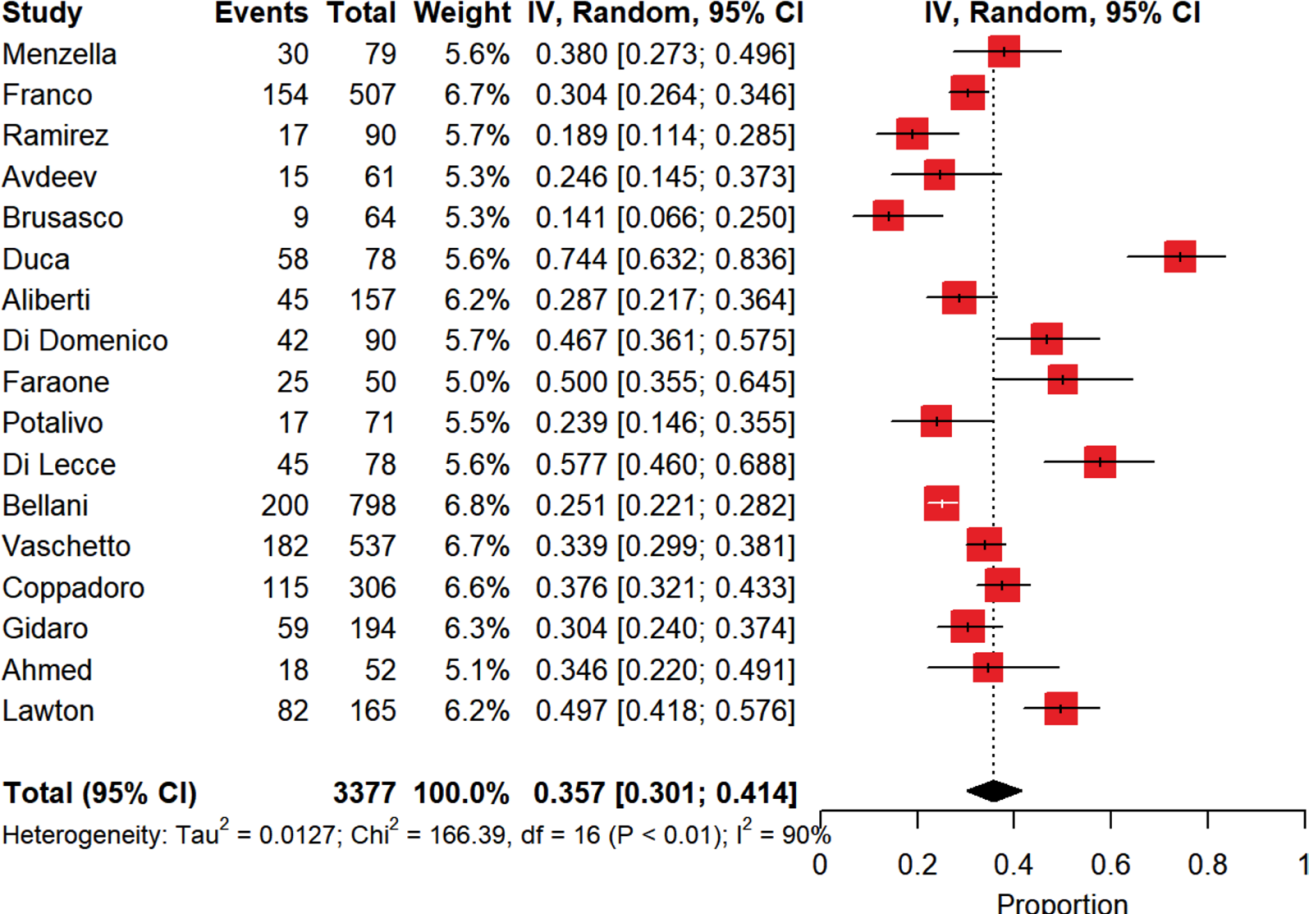
Pooled overall intra-hospital mortality of patients assisted through noninvasive respiratory support outside the intensive care unit. The vertical dotted line refers to the summary estimate for overall intra-hospital mortality of patients assisted through noninvasive respiratory support outside intensive care unit. Red squares indicate the individual study estimates of the overall intra-hospital mortality of patients assisted through noninvasive respiratory support outside the intensive care unit, whereas the black horizontal lines indicate the 95% confidence interval of single studies. The diamond refers to the summary estimate with 95% confidence interval

Figure 1 depicts the overall intra-hospital mortality in patients noninvasively ventilated outside the ICU. Overall intra-hospital mortality rate was 36% [30–41%] in COVID-19 patients who received NIRS outside the ICU, with a high between-study heterogeneity (*p* < 0.0001, *I*^2^ = 90.4%).

**Fig. 2:**
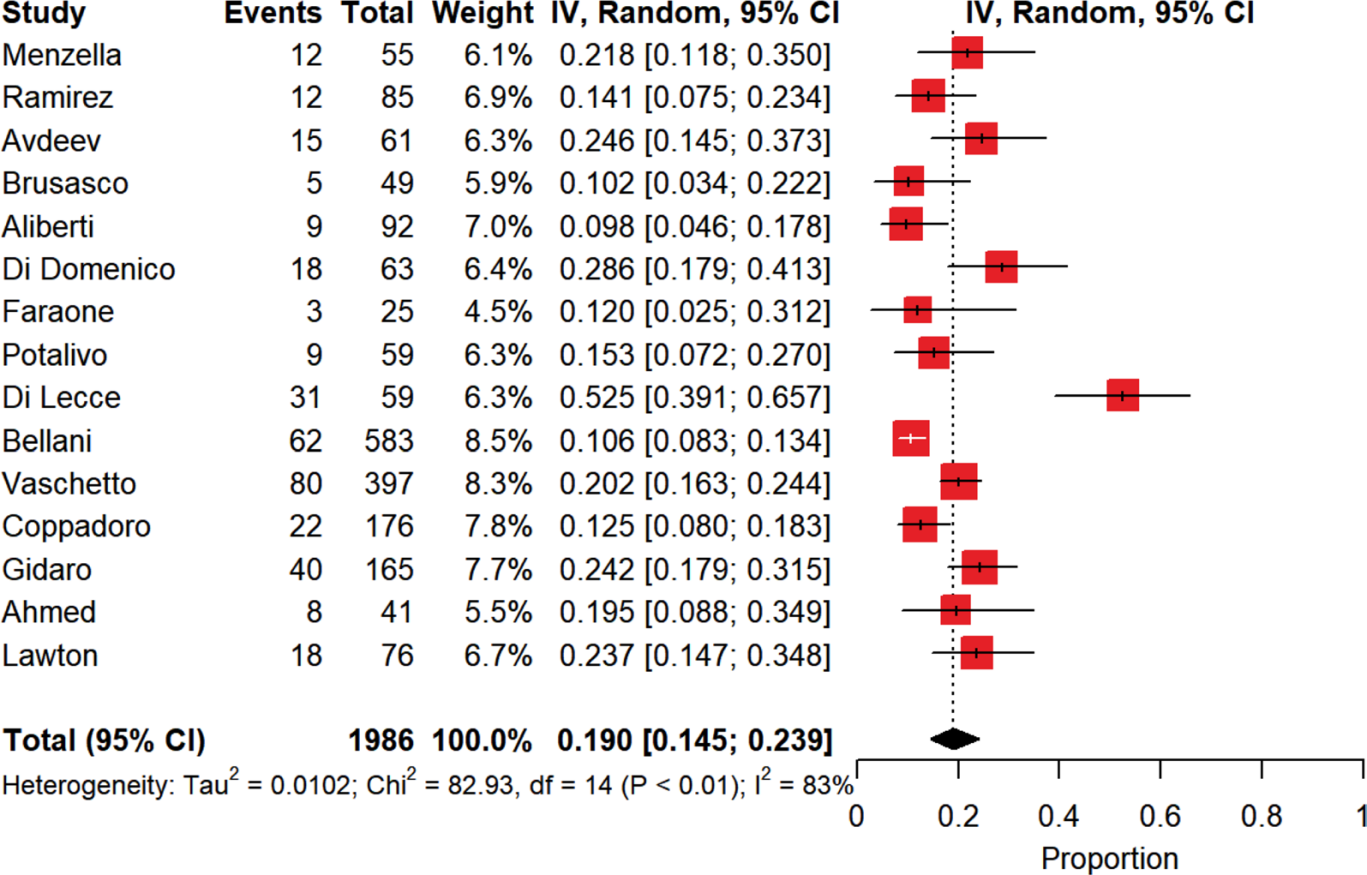
Pooled intra-hospital mortality of patients assisted through noninvasive respiratory support outside the intensive care unit, net of the patients subjected to limitations of care. The vertical dotted line refers to the summary estimate for intra-hospital mortality of patients assisted through noninvasive respiratory support outside intensive care unit, excluding the patients subjected to limitations of care. Red squares indicate the individual study estimates of the overall intra-hospital mortality of patients assisted through noninvasive respiratory support outside the intensive care unit, excluding the patients subjected to limitations of care, whereas the black horizontal lines indicate the 95% confidence interval of single studies. The diamond refers to the summary estimate with 95% confidence interval

As depicted in Fig. 2, when patients subjected to DNI orders were excluded from the global population of patients assisted by NIRS outside the ICU, the pooled intra-hospital mortality was 19% [15–24%] with a high between-study heterogeneity (*p* < 0.01, *I*^2^ = 83%).

**Fig. 3:**
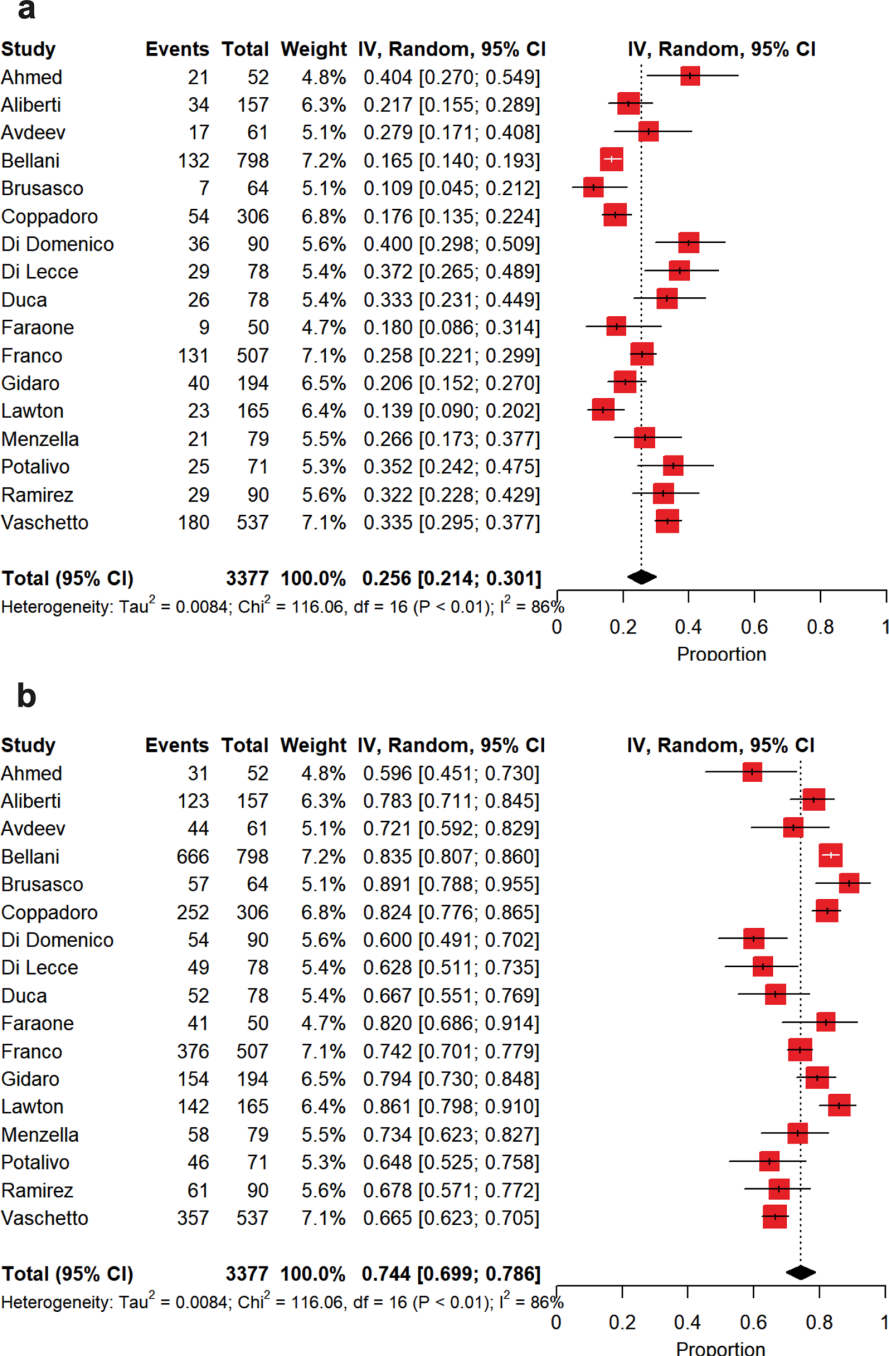
Summary estimates of intubated patients following noninvasive respiratory support failure and of those patients who continued noninvasive respiratory support and did not experience intubation. **a** Summary estimate of intubated patients following noninvasive respiratory support failure. The vertical dotted line refers to the summary estimate of intubation in patients who failed noninvasive respiratory support. Red squares indicate the individual study estimates of the intubated patients following noninvasive respiratory support failure, whereas the black horizontal lines indicate the 95% confidence interval of single studies. The diamond refers to the summary estimate with 95% confidence interval. **b** Summary estimate of patients with noninvasive respiratory support who did not experience intubation. The vertical dotted line refers to the summary estimate of patients who continued noninvasive respiratory support and were not intubated. Red squares indicate the individual study estimates of patients who continued noninvasive respiratory support and were not intubated, whereas the black horizontal lines indicate the 95% confidence interval of single studies. The diamond refers to the summary estimate with 95% confidence interval

The estimate of intubation rate is shown in Fig. 3a. Pooled intubation estimate was 26% [21–30%], with a high between-study heterogeneity (*p* < 0.0001, *I*^2^ = 86.2%). In this case, the source of heterogeneity was only ascribed to PaO_2_/FiO_2_ on admission (*p* < 0.0001, *I*^2^ = 73.4%). The causes of IMV onset and the intubation criteria are described in Additional file 3-Tables 7 and 8. Among the included studies, 8 investigations attributed refractory hypoxemia to the cause of intubation.

**Fig. 4:**
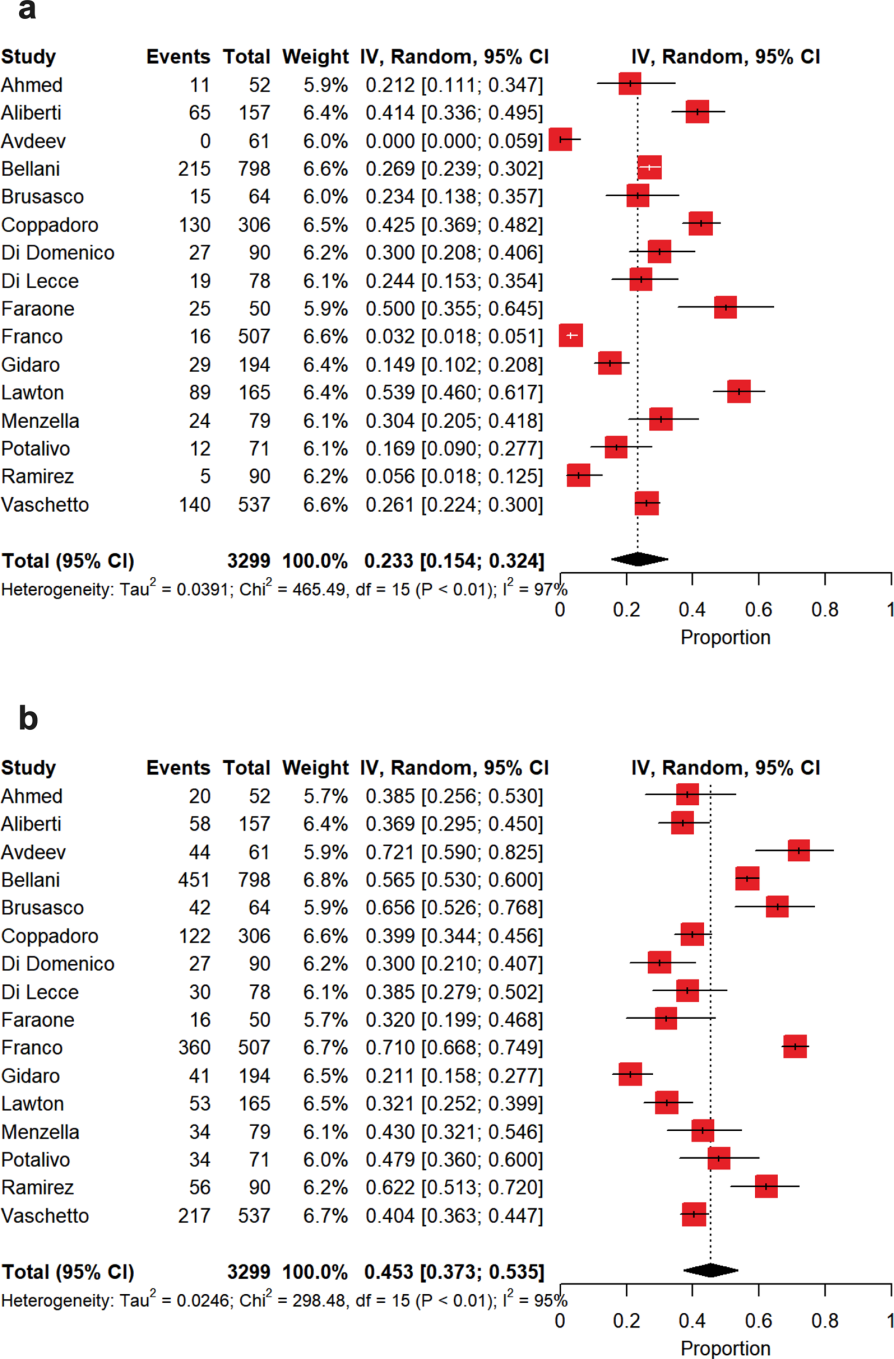
Summary estimates of patients with limitations of care and of those under *‘full treatment’* who were not intubated. **a** Summary estimate of nonintubated patients with limitations of care. The vertical dotted line refers to the summary estimate of patients with limitations of care. Red squares indicate the individual study estimates of the patients with limitations of care, whereas the black horizontal lines indicate the 95% confidence interval of single studies. The diamond refers to the summary estimate with 95% confidence interval. **b** Summary estimate of patients under *‘full treatment’* who were not intubated. The vertical dotted line refers to the summary estimate of patients under *‘full treatment’* who were not intubated. Red squares indicate the individual study estimates of patients under *‘full treatment’* who were not intubated, whereas the black horizontal lines indicate the 95% confidence interval of single studies. The diamond refers to the summary estimate with 95% confidence interval

**Fig. 5:**
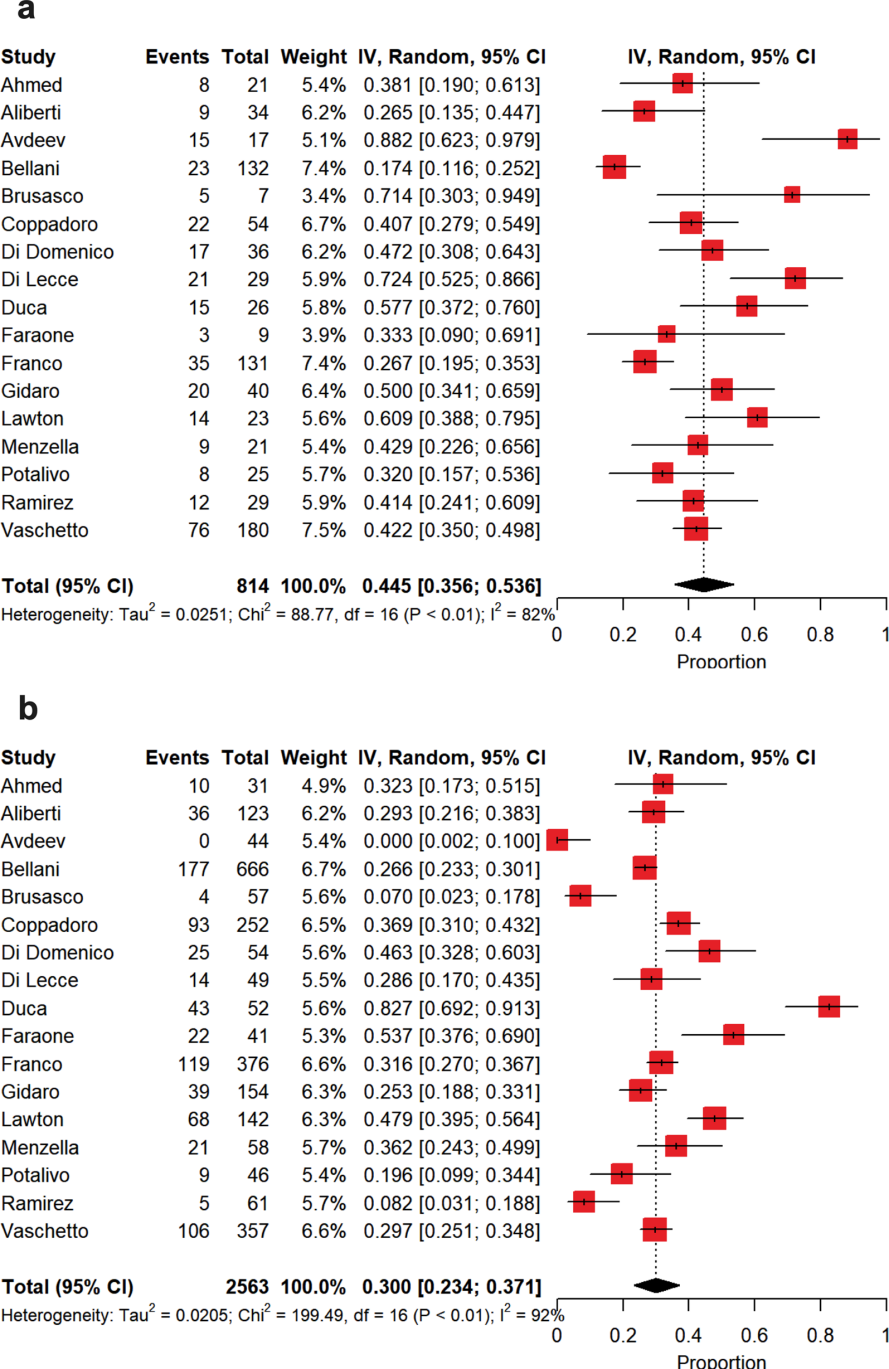
Summary estimates of intra-hospital mortality observed in intubated patients following noninvasive respiratory support failure and of that reported in patients who continued noninvasive respiratory support and did not experience intubation. **a** Summary estimate of intra-hospital mortality observed in intubated patients following noninvasive respiratory support failure. The vertical dotted line refers to the summary estimate of intra-hospital mortality observed in intubated patients who failed noninvasive respiratory support. Red squares indicate the individual study estimates of intra-hospital mortality observed in intubated patients following noninvasive respiratory support failure, whereas the black horizontal lines indicate the 95% confidence interval of single studies. The diamond refers to the summary estimate with 95% confidence interval. **b** Summary estimate of intra-hospital mortality of patients with noninvasive respiratory support who did not experience intubation. The vertical dotted line refers to the summary estimate of intra-hospital mortality of patients with noninvasive ventilation who did not experience intubation. Red squares indicate the individual study estimates of intra-hospital mortality of patients with noninvasive respiratory support who did not experience intubation, whereas the black horizontal lines indicate the 95% confidence interval of single studies. The diamond refers to the summary estimate with 95% confidence interval

**Fig. 6:**
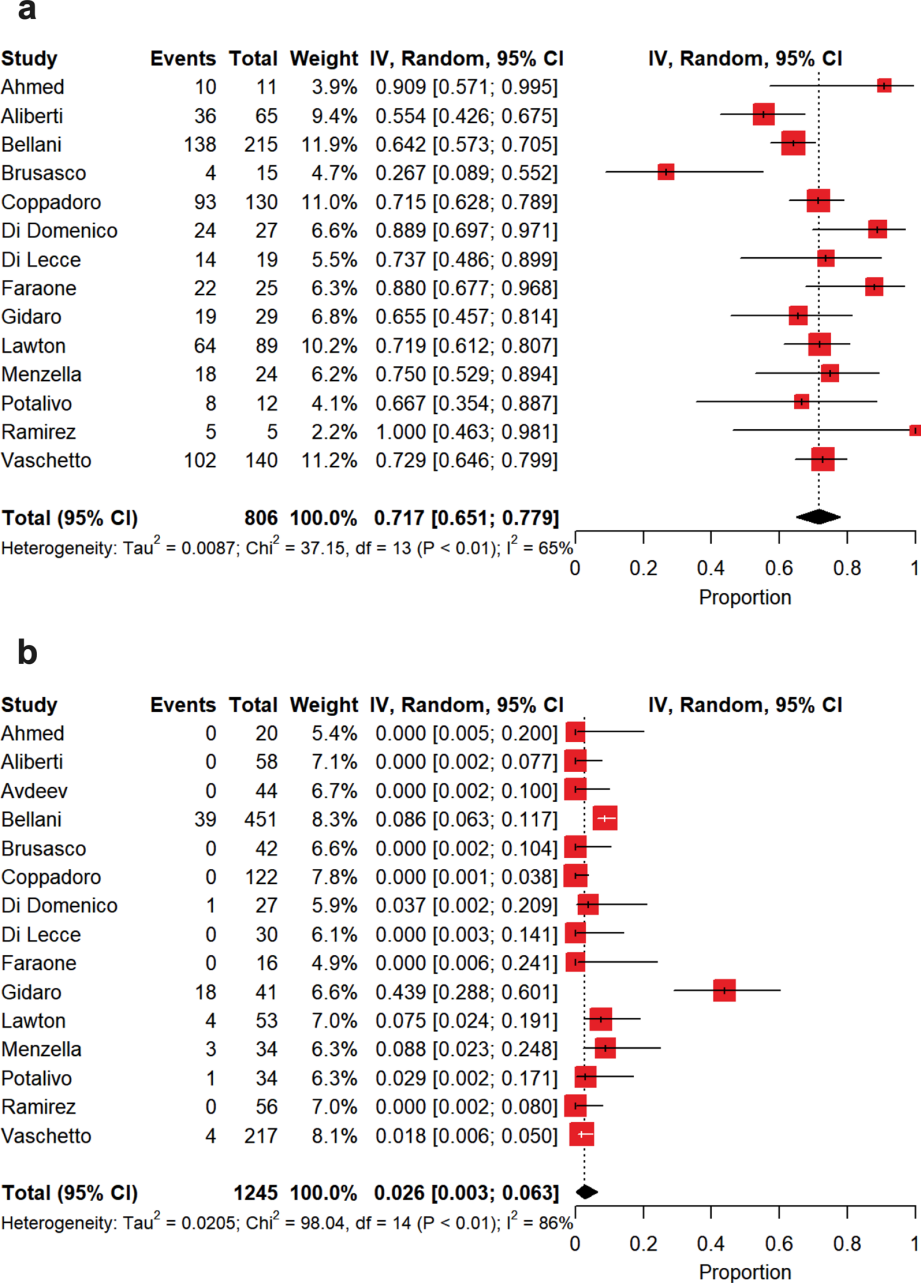
Summary estimates of intra-hospital mortality observed in patients with limitations of care and of that reported in patients under *‘full treatment’* who were not intubated. **a** Summary estimate of intra-hospital mortality observed in patients with limitations of care. The vertical dotted line refers to the summary estimate of intra-hospital mortality observed in patients with limitations of care. Red squares indicate the individual study estimates of intra-hospital mortality observed in patients with limitations of care, whereas the black horizontal lines indicate the 95% confidence interval of single studies. The diamond refers to the summary estimate with 95% confidence interval. **b** Summary estimate of intra-hospital mortality observed in patients under *‘full treatment’* who were not intubated. The vertical dotted line refers to summary estimate of intra-hospital mortality observed in patients under *‘full treatment’* who were not intubated. Red squares indicate individual study estimates of intra-hospital mortality observed in patients under *‘full treatment’* who were not intubated, whereas the black horizontal lines indicate the 95% confidence interval of single studies. The diamond refers to the summary estimate with 95% confidence interval

The summary estimate of nonintubated patients is depicted in Fig. 3b. Patients were not intubated in 74% [70–79%] of the cases, with a high between-study heterogeneity (*p* < 0.0001, *I*^2^ = 86.2%). Also in this case, this heterogeneity was only due to PaO_2_/FiO_2_ on admission (*p* < 0.0001, *I*^2^ = 73.4%). Among patients who did not experience intubation, a DNI order was expressed in a summary estimate of 23% [15–32%] of the cases (Fig. 4a), whereas patients were deemed as deserving ‘*full treatment*’ in 45% [37–54%] of the cases (Fig. 4b), with a high heterogeneity for both (DNI, *p* < 0.0001, *I*^2^ = 96.8%; ‘*full treatment’*, *p* < 0.0001, *I*^2^ = 95.0%). As depicted in Fig. 5a, in patients who failed NIV and were subsequently intubated, intra-hospital mortality reached 45% [36–54%], while in those subjects under NIRS who did not experience IMV intra-hospital mortality was of 30% [23–37%] (Fig. 5b), with high between-study heterogeneities in both the cases (intubation, *p* < 0.0001, *I*^2^ = 82.0%; nonintubation, *p* < 0.0001, *I*^2^ = 92.0%). In the subset of NIRS patients with DNI orders (Fig. 6a), intra-hospital mortality was of 72% [65–78%], with a moderate between-study heterogeneity (*p* < 0.0004, *I*^2^ = 65.0%), while in those under NIRS deserving ‘*full treatment*’ (Fig. 6b) intra-hospital mortality reached 2.6% [0.3–6.3%], with a high between-study heterogeneity (*p* < 0.0001, *I*^2^ = 85.7%).

In Additional file 3-Table 9, hospital length of stay, NIRS and IMV duration, along with time lag between NIRS and IMV onset were reported. Interestingly, NIRS-to-IMV time lag varied from a mean value of 72 h to a mean value of 137 h (1524/3377 patients).

## Discussion

In the present systematic review and meta-analysis patients admitted for COVID-19 and requiring NIRS outside the ICU were characterized by an overall intra-hospital mortality of 36%.

During COVID-19 outbreak, NIRS was demonstrated to be feasible both in- and outside ICU, in a percentage of patients ranging between 11 and 62% [3, 8, 9, 35, 36]. Despite the lack of a strong recommendation in pandemic viral illness [15], several observational studies suggest that the application of NIRS was clinically useful in stabilizing the clinical course of patients with mild-to-moderate ARF COVID-19 related [3, 9]. However, due to a potential imbalance between the exceptional demand for ventilatory assistance during COVID-19 pandemic and hospital surge capacity, one might suppose that NIRS practiced outside ICU would be characterized by an increase in intra-hospital mortality compared to NIRS applied in the ICU for COVID-19 patients with ARF. Indeed, data from our 3377 patients showed that (1) NIRS outside the ICU was feasible in the COVID-19 pandemic scenario and (2) in our global patients’ population receiving NIRS outside the ICU, the pooled intra-hospital mortality of 19%, net of patients subjected to DNI orders, was quite similar to intra-hospital mortality observed in the helmet NIRS group from a recent randomized-controlled trial, conducted in COVID-19 patients admitted to ICU [38].

In our population pooled intubation rate was of 26%. This finding kept with IMV onset reported in severe acute respiratory syndrome [39], but was lower than that described for H1N1 pneumonia [40], and middle eastern respiratory syndrome outbreak [41]. In our context, the most reported cause of intubation was refractory hypoxemia, when the reasons for IMV commencement were described, with an average NIRS-to-IMV time lag varying from a minimum of 55 h to a maximum of 137 h, respectively, when reported. Once intubated, in this subset of patients who experienced NIRS failure, we observed a pooled intra-hospital mortality of 45%, consistent with that observed in intubated ARDS patients who failed NIRS [37]. In interpreting our data, it is worth to consider that an undue prolongation of NIRS with a consequent delayed intubation probably played a key role in the lung injury progression, as described by patient self-induced lung injury theory [42].

The rate of DNI order application has increased over time in the last two decades, reaching 32% in patients admitted for ARF undergoing NIRS or high flow oxygen therapy [43], in nonpandemic context. In this subset of patients, a pooled survival of 56% at hospital discharge has been reported regardless of whether patients were managed in the ICU or hospital ward [44]. However, the DNI order decision-making process is particularly tricky because it is affected by demographic and clinical factors, i.e., age and illness severity, along with patient/family involvement [43]. According to our findings, in the COVID-19 pandemic context, the summary estimate of the patients, in whom a DNI decision was pursued, was 23% with a pooled intra-hospital mortality of 72%. It is worth to point out that our data were obtained during the first wave of COVID-19 outbreak, with the well-described concerns of hospital and ICU surge capacity [7, 45].

Among the variables investigated, PaO_2_/FiO_2_ on admission was the main factor sustaining the between-study heterogeneities of the investigated outcomes. These data suggest that, in our context, there was most likely a great variability in the modalities of proceeding toward intubation or continuing NIRS. In this regard, other factors, such as the availability of resources and the strategy of their allocation, might have adversely influenced the process of care [7, 45].

As a clinical implication, our findings, in agreement with previous suggestions [47], support the use of NIV for hypoxemic ARF due to COVID-19 also outside ICU, in the intermediate care unit setting.

The present investigation has several limitations requiring to be discussed. The enrolled studies were mainly retrospective investigations conducted during the first wave of COVID-19 pandemic from the end of February to the end of May 2020. Accordingly, it is worth to take into account the critical issues of the specific historical moment, characterized by the crisis of the hospital surge capacity response and the lack of a well-defined therapeutic approach. We could not provide insights on the modalities of NIRS application, conduction, and monitoring in the different settings explored because of the paucity of data retrieved. The leading part of the included studies was carried out in Italy. Thus, our conclusions cannot be generalized to other countries with different policies, practices, medical ethics, social attitudes, cultures, and religions [48–51]. We could not draw any conclusions about the efficacy of NIRS in curbing the overall intra-hospital mortality in the light of our data. Indeed, the present analysis was conducted on data retrieved exclusively from retrospective and prospective, nonrandomized investigations, accounting for indication bias and confounding. We included 3 pre-print investigations [32–34] in our analysis because of the relatively small number of studies enrolled at the time of search closure (end of February 21). This latter aspect along with the high between-study heterogeneity, the lack of a specific time point of intra-hospital mortality observation, and, in some cases, the poor data reporting could limit the possibility to draw definitive conclusions from our data.

## Conclusions

This systematic review and meta-analysis summarized the evidence reported from the first wave of COVID-19 outbreak on the incidence of overall intra-hospital mortality in hospitalized patients undergoing NIRS outside the ICU. Despite the concerns arising from the crisis of hospital surge capacity response and the lack of a clinically effective therapy, delivering NIRS outside the ICU revealed overall as a feasible strategy to cope with the massive demand of ventilatory assistance even for those patients with care limitations. Our findings require to be confirmed in future investigations addressing the same topic over the following waves of COVID-19 outbreak.

## Supplementary Information

**Additional file 1**. Search strategy of electronic database.

**Additional file 2**. Enrollment flow diagram.

**Additional file 3**. Characteristics of the enrolled investigations, overall clinical characteristics of the populations investigated in the enrolled studies, and list of studies excluded after reading the full text.

**Additional file 4**. Methodological quality of the included investigations.

## Data Availability

The datasets used and/or analyzed during the current study are available from the corresponding author on reasonable request.

## Abbreviations

ARDS: Acute respiratory distress syndrome
ARF: Acute respiratory failure
CPAP: Continuous positive airway pressure
CI: Confidence interval
COVID-19: Coronavirus-19 disease
DNI: Do-not-intubate
ICU: Intensive care unit
IMV: Invasive mechanical ventilation
NIRS: Noninvasive respiratory support
PaO_2_/FiO_2_: Arterial oxygen tension on inspired oxygen fraction ratio
SARS-CoV-2: Severe acute respiratory syndrome related to novel coronavirus
