# Supplementary material for: Noninvasive respiratory support outside the intensive care unit for acute respiratory failure related to coronavirus-19 disease: a systematic review and meta-analysis
Source: Crit Care. 2021 Jul 30;25:268. doi: 10.1186/s13054-021-03697-0 (PMC8324455; doi:10.1186/s13054-021-03697-0)
Supplement: Supplementary file 1 — Additional file 1. Search strategy of electronic database. [file 13054_2021_3697_MOESM1_ESM.docx]

**Additional file 1.** Search strategy of electronic databases.

| **Keywords** | | |
| --- | --- | --- |
| COVID-19 OR Novel Coronavirus 2019 OR SARS-CoV-2 OR SARS-CoV-19 OR Severe Acute Respiratory Syndrome OR Severe Acute Respiratory Syndrome Coronavirus Related | AND | Positive Pressure Respiration OR NIV OR Non Invasive Ventilation OR CPAP OR Continuous Positive Airway Pressure OR non invasive positive pressure respiration OR NIPPV OR NRS OR Non Invasive Respiratory Support OR Intermittent Positive Pressure Ventilation |

**Pubmed**

("COVID-19"[Title/Abstract] OR "novel coronavirus 2019"[Title/Abstract] OR "SARS-CoV-2"[Title/Abstract] OR ((("severe acute respiratory syndrome"[MeSH Terms] OR ("severe"[All Fields] AND "acute"[All Fields] AND "respiratory"[All Fields] AND "syndrome"[All Fields]) OR "severe acute respiratory syndrome"[All Fields]) AND ("novel"[All Fields] OR "novel s"[All Fields] OR "novels"[All Fields])) AND "coronavirus related"[Title/Abstract]) OR "sars covid 19"[Title/Abstract] OR "sars-covid-2"[Title/Abstract] OR "coronavirus"[MeSH Terms]) AND ("positive pressure respiration"[MeSH Terms] OR ("NIV"[Title/Abstract] OR "non invasive ventilation"[Title/Abstract] OR "CPAP"[Title/Abstract] OR "continuous positive airway pressure"[Title/Abstract] OR ("non-invasive"[All Fields] AND "positive pressure respiration"[Title/Abstract]) OR "NIPPV"[Title/Abstract] OR "NRS"[Title/Abstract] OR "non invasive respiratory support"[Title/Abstract]) OR (("non"[All Fields] AND ("invasibility"[All Fields] OR "invasible"[All Fields] OR "invasion"[All Fields] OR "invasions"[All Fields] OR "invasive"[All Fields] OR "invasively"[All Fields] OR "invasiveness"[All Fields] OR "invasives"[All Fields] OR "invasivity"[All Fields])) AND ("positive pressure respiration"[MeSH Terms] OR "intermittent positive pressure ventilation"[MeSH Terms])))

#448

**Embase**

‘Coronavirus disease 2019’ OR ‘SARS-related coronavirus 2019’ OR ‘SARS-related coronavirus’ AND ‘continuous positive airway pressure' OR 'noninvasive positive pressure ventilation' OR 'CPAP device' OR 'noninvasive ventilation'

#279

**Cochrane**

‘Noninvasive positive pressure respiration’ OR ‘continuous positive airway pressure’ OR ‘non-invasive ventilation’ OR ‘CPAP or NIV’ AND ‘COVID-19’ OR ‘novel coronavirus’ OR ‘SARS-COVID-2’ OR ‘severe acute respiratory syndrome coronavirus 2’ OR ‘severe acute respiratory failure novel coronavirus-related’

#318

**medRxiv**

‘COVID-19’ AND ‘noninvasive ventilation’

#820

**bioRxiv**

‘COVID-19’ AND ‘noninvasive ventilation’

#91
