# Supplementary figures and images for: Noninvasive respiratory support outside the intensive care unit for acute respiratory failure related to coronavirus-19 disease: a systematic review and meta-analysis
Source: Crit Care. 2021 Jul 30;25:268. doi: 10.1186/s13054-021-03697-0 (PMC8324455; doi:10.1186/s13054-021-03697-0)

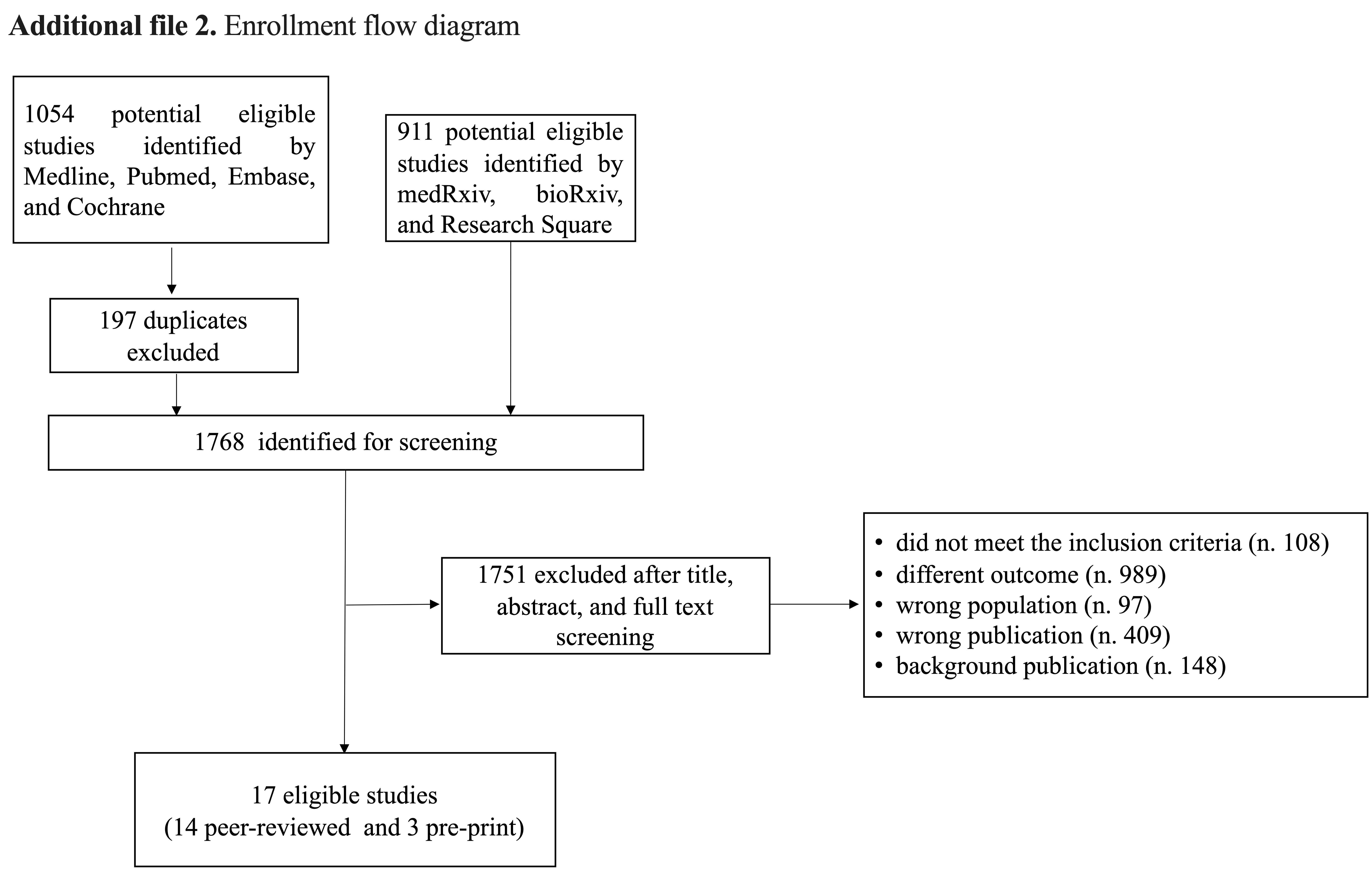

Supplement: Supplementary file 2 — Additional file 2. Enrollment flow diagram. [file 13054_2021_3697_MOESM2_ESM.tiff]

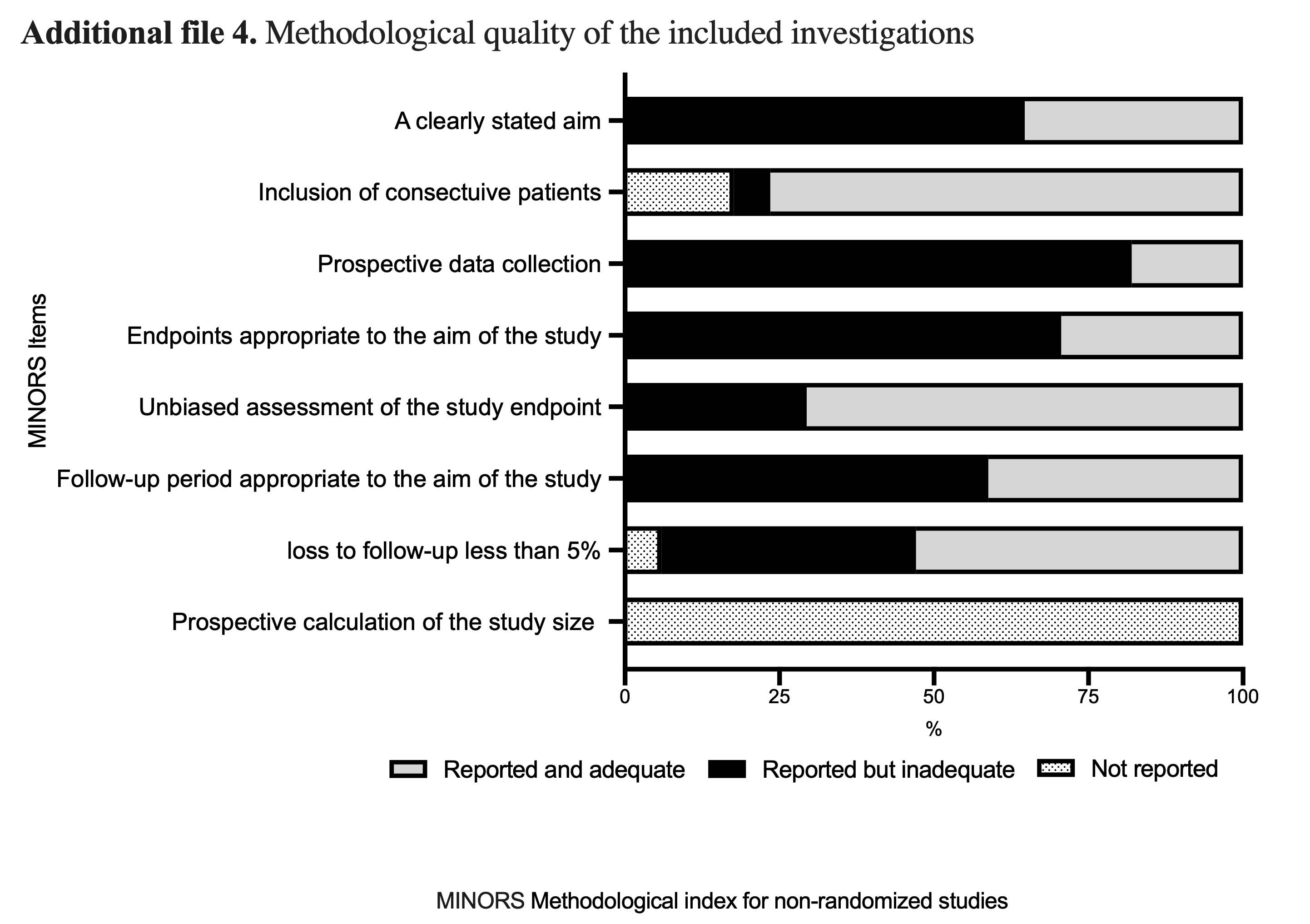

Supplement: Supplementary file 4 — Additional file 4. Methodological quality of the included investigations. [file 13054_2021_3697_MOESM4_ESM.tiff]
