# Supplementary material for: Noninvasive respiratory support outside the intensive care unit for acute respiratory failure related to coronavirus-19 disease: a systematic review and meta-analysis
Source: Crit Care. 2021 Jul 30;25:268. doi: 10.1186/s13054-021-03697-0 (PMC8324455; doi:10.1186/s13054-021-03697-0)
Supplement: Supplementary file 3 — Additional file 3. Characteristics of the enrolled investigations, overall clinical characteristics of the populations investigated in the enrolled studies, and list of studies excluded after reading the full text. [file 13054_2021_3697_MOESM3_ESM.docx]

| **Additional file Table 1.** Characteristics of included studies. | | | | | |  |  |
| --- | --- | --- | --- | --- | --- | --- | --- |
| **Study** | **Study design** | **Centers** | **Location** | **Setting** | **Inclusion start date** | **Inclusion end date** | **Patients with suspect of COVID-19 infection on hospital admission (No)** |
|  |  |  |  |  |  |  |  |
| Menzella et al. | Retrospective | Single center | Italy | PU | 10-March-20 | 14-April-20 | 79 |
| Franco et al. | Prospective | Multicenter | Italy | PU | 1-March-20 | 10-May-20 | 704 |
| Ramirez et al. | Prospective | Single center | Italy | IMU | 25-February-20 | 15-April-20 | 90 |
| Avdeev et al. | Retrospective | Multicenter | Russia | C19CU | 8-April-20 | 10-June-20 | 297 |
| Brusasco et al. | Retrospective | Single center | Italy | SICU | 16-March-20 | 12-April-20 | 258 |
| Duca et al. | Retrospective | Single center | Italy | ED | 29-February-20 | 10-March-20 | 611 |
| Aliberti et al. | Prospective | Multicenter | Italy | HDU | 7-March-20 | 21-April-20 | 157 |
| Di Domenico et al. | Retrospective | Single center | Italy | ED/RICU | 29-February-20 | 19-March-20 | 776 |
| Faraone et al. | Retrospective | Single center | Italy | C19CU | 6-March-2020 | 7-May-2020 | 143 |
| Potalivo et al. | Retrospective | Single center | Italy | C19CU | 26-February-20 | 18-April-20 | 1424 |
| Di Lecce et al. | Retrospective | Single center | Italy | RICU | 11-March-2020 | 31-May-2020 | 97 |
| Bellani et al. | Retrospective | Multicenter | Italy | C19CU | 26-March-2020* | 31-March-2020* | 8753 |
| Vaschetto et al. | Retrospective | Multicenter | Italy | RICU | 1-March-20 | 15-April-20 | 2845 |
| Coppadoro et al. | Retrospective | Multicenter | Italy | C19CU | 3-March-20 | 3-April-20 | 306 |
| Gidaro et al. | Retrospective | Single center | Italy | C19CU | 21-February-2020 | 6-March-2020 | 1016 |
| Ahmed et al. | Retrospective | Single center | United Kingdom | RICU | 1-March-20 | 30-April-20 | 89 |
| Lawton et al. | Retrospective | Single center | United Kingdom | C19CU | 26-February-20 | 1-May-20 | 559 |
|  |  |  |  |  |  |  |  |

COVID-19, coronavirus-19 disease PU, pulmonology unit; IMU, internal medicine unit; C19CU, COVID-19 care unit; SICU, sub-intensive care unit; RICU, respiratory intermediate care unit; ED, emergency department; HDU, high-dependency unit. *single day study, COVID-19, novel coronavirus 19 disease.

| **Study** | **A clearly stated aim** | **Inclusion of consecutive patients** | **Prospective data collection** | **Endpoints appropriate to the aim of the study** | **Unbiased assessment of the study endpoint** | **Follow-up period appropriate to the aim of the study** | **Loss to follow-up less than 5%** | **Prospective calculation of the study size** |
| --- | --- | --- | --- | --- | --- | --- | --- | --- |
|  |  |  |  |  |  |  |  |  |
| Menzella et al. | 1 | 2 | 1 | 1 | 1 | 1 | 1 | 1 |
| Franco et al. | 1 | 2 | 2 | 1 | 1 | 1 | 1 | 1 |
| Ramirez et al. | 1 | 2 | 2 | 1 | 2 | 2 | 2 | 1 |
| Avdeev et al. | 1 | 2 | 1 | 1 | 2 | 2 | 2 | 1 |
| Brusasco et al. | 2 | 2 | 1 | 2 | 2 | 2 | 2 | 1 |
| Duca et al. | 1 | 2 | 1 | 1 | 2 | 2 | 2 | 1 |
| Aliberti et al. | 2 | 2 | 2 | 2 | 2 | 1 | 1 | 1 |
| Di Domenico et al. | 1 | 2 | 1 | 1 | 2 | 2 | 2 | 1 |
| Faraone et al. | 1 | 2 | 1 | 1 | 2 | 1 | 2 | 1 |
| Potalivo et al. | 1 | 2 | 1 | 1 | 2 | 1 | 2 | 1 |
| Di Lecce et al. | 1 | 0 | 1 | 1 | 1 | 1 | 1 | 1 |
| Bellani et al. | 2 | 1 | 1 | 1 | 1 | 1 | 0 | 1 |
| Vaschetto et al. | 1 | 2 | 1 | 1 | 2 | 2 | 1 | 1 |
| Coppadoro et al. | 2 | 2 | 1 | 2 | 2 | 2 | 2 | 1 |
| Gidaro et al. | 2 | 0 | 1 | 2 | 2 | 1 | 1 | 1 |
| Ahmed et al. | 2 | 0 | 1 | 2 | 2 | 1 | 1 | 1 |
| Lawton et al. | 1 | 2 | 1 | 1 | 1 | 1 | 2 | 1 |
|  |  |  |  |  |  |  |  |  |

| **Additional file Table 2.**  Methodological index for non-randomized studies (MINORS) tool criteria and scores. |
| --- |

MINORS, methodological index for non-randomized studies. Each item was scored as follows: 0 (not reported), 1 (reported but inadequate), or 2 (reported and adequate).

| **Additional file Table 3.** Patients’ demographic characteristics and comorbidities. | | | | | | | | | | | | |
| --- | --- | --- | --- | --- | --- | --- | --- | --- | --- | --- | --- | --- |
| **Study** | **Patients with NIRS No.** | **Male**  **No. (%)** | **Age (years)** | **BMI (kg/m^2^)** | **DNI**  **No. (%)** | **Charlson index** | **Hypertension No. (%)** | **Respiratory No. (%)** | **Cardiovascular No. (%)** | **Kidney**  **No. (%)** | **Diabetes No. (%)** | **Active Smoke No. (%)** |
|  |  |  |  |  |  |  |  |  |  |  |  |  |
| Menzella et al. | 79 | 56 (71) | 67±11 | 29.7±5.2 | 24 | 3±2 | 48 (61) | 12 (15) | 24 (30) | 6 (8) | 18 (23) | 3 (4) |
| Franco et al. | 507 | 350 (69) | 69±13 | - | 16* | - | 237 (47) | 37 (7) | 76 (15) | 22 (4) | 93 (18) | - |
| Ramirez et al. | 90 | 72 (80) | 62±14 | 28.0±5* | 5 | - | 28 (33)* | 5 (6)* | 28 (33)* | 4 (5)* | 16 (19)* | - |
| Avdeev et al. | 61 | 37 (61) | 62±13 | 31.9±4.8 | 0 | - | 29 (48) | - | 3 (5) | 2 (3) | 8 (13) | 30 (50) |
| Brusasco et al. | 64 | 41 (64) | 60 | - | 15 | - | 22 (34) | 6 (9) | 10 (16) | 10 (16) | 12 (19) | 11 (17) |
| Duca et al. | 78 | 66 (85) | 70±13.5 | - | - | 3±2 | 44 (56) | 6 (8) | 8 (10) | 1 (1) | 19 (24) | - |
| Aliberti et al. | 157 | 117 (74.5) | 65±15 | 27.3±4.0 | 65 | - | 69 (44) | 13 (8) | 98 (62) | 9 (6) | 36 (23) | 27 (17) |
| Di Domenico et al. | 90 | 72 (80) | 65±14 | 29.2±4.42 | 27 | - | 50 (55) | 10 (11) | 20 (22) | 8 (8) | 19 (21) | 14 (15) |
| Faraone et al. | 50 | 33 (66) | 75 ±11 | - | 25 | - | 28 (56) | 10 (20) | 36 (72) | 10 (20) | 12 (24) | - |
| Potalivo et al. | 71 | 56 (78) | 65±11 | - | 12 | 2.4±1.4* | 30 (42) | - | - | - | - | - |
| Di Lecce et al. | 78 | 69 (88) | 70±14 | 28.0±5 | 19 | 4±3 | 63 (80) | 8 (10) | 53 (68) | 46 (59) | 30 (38) | 4 (5) |
| Bellani et al. | 798 | 595 (75) | 67±11.9 | 27.4±4.5* | 215 | - | 438 (55) | 76 (10) | 200 (25) | 32 (4) | 160 (20) | 36 (5) |
| Vaschetto et al. | 537 | 391 (73) | 68±12 | 28.0±4.5* | 140 | 1±1 | 278 (52) | - | 66 (12) | - | 138 (26) | - |
| Coppadoro et al. | 306 | 236 (77) | 66±11 | 27.0±6.0 | 130 | - | 159 (52) | 23 (8) | 68 (22) | 18 (6) | 62 (20) | - |
| Gidaro et al. | 194 | 139 (72) | 64±15 | - | 29 | 3±2 | - | - | - | - | - | - |
| Ahmed et al. | 52 | 34 (65) | 61±12 | 28.6±6.2 | 11 | - | 22 (42) | 19 (37) | 13 (25) | 9 (17) | 13 (25) | - |
| Lawton et al. | 165 | 96 (58) | 62±15 | 30±7.2 | 89 | - | - | - | - | - | - | - |
|  |  |  |  |  |  |  |  |  |  |  |  |  |

Data are presented as number and percentage or mean and standard deviation. NIRS, noninvasive respiratory support; BMI, body mass index; DNI, “do-not-intubate” orders *, not available for the whole study population.

| **Additional file Table 4.** Laboratory test findings, oxygenation, and respiratory rate at hospital admission. | | | | | | | | | | | | | | | | |
| --- | --- | --- | --- | --- | --- | --- | --- | --- | --- | --- | --- | --- | --- | --- | --- | --- |
| **Study** | **Patients enrolled with NIRS No.** | **CRP (mg/dl)** | **LDH (U/l)** | **D-dimer (μgFEU/l)** | **Creatinine (mg/dl)** | **Ferritin (ng/ml)** | **IL-6 (pg/ml)** | | **Lymphocyte count (x10^3^/μl)** | **White cell count (x10^3^/μl)** | | **PaO_2_/FiO_2_ (mmHg)** | | | **Respiratory rate (breaths/min)** | |
|  |  |  |  |  |  |  |  |  | | |  | |  |  | |  |
| Menzella et al. | 79 | 12±7 | 688±245 | 775±917 | - | 689±684 | 147±180 | 0.8±0.4 | | | 6.4±2.7 | | 120±42 | 25±5 | |  |
| Franco et al. | 507 | - | - | - | - | - | - | - | | | - | | 147±82 | 29±7 | |  |
| Ramirez et al. | 90 | 14±10* | 481±146* | - | 1±0.2* | - | - | 0.8±0.4* | | | 8.1±4.6* | | 92±5 | 30±8 | |  |
| Avdeev et al. | 61 | 14±10 | - | 1064±538 | - | - | - | 0.9±0.5 | | | 7.6±3.3 | | 165±52 | 24±6 | |  |
| Brusasco et al. | 64 | 7±5 | 357±134 | 1849±2915 | - | 989±788 | - | 1.2±1.9 | | | 9.0±4.7* | | 124±41 | 33±5 | |  |
| Duca et al. | 78 | - | - | - | - | - | - | - | | | - | | 143±68 | - | |  |
| Aliberti et al. | 157 | 15±19 | - | - | - | - | - | - | | | - | | 148±80 | - | |  |
| Di Domenico et al. | 90 | 12* | - | - | - | - | - | 1.2* | | | - | | - | - | |  |
| Faraone et al. | 50 | - | - | - | - | - | - | - | | | - | | 130±64 | - | |  |
| Potalivo et al. | 71 | - | - | - | - | - | - | - | | | - | | 106±5 | - | |  |
| Di Lecce et al. | 78 | 12±8 | 334±109 | 2429±3794 | 1±1 | 838±915 | - | 0.9±0.7 | | | 7.8±3.8 | | 191±114 | - | |  |
| Bellani et al. | 798 | 11±8 | - | - | 1±0.8* | - | - | - | | | 10.2±8.9* | | 162±102* | - | |  |
| Vaschetto et al. | 537 | 11±8 | 585±279 | 1118±1002 | 1±0.4* | 1087±801* | - | 0.8±0.4* | | | 7.2±3.3* | | 112±64* | 27±7* | |  |
| Coppadoro et al. | 306 | 11±9 | 425±143 | - | 1±0.4 | - | - | - | | | 7.7±3.4 | | 131±71 | 27±7 | |  |
| Gidaro et al. | 194 | 17±10 | - | 842.2±217.2 | - | - | 139±377 | 1.1±2 | | | 7.0±8.8 | | 220±101 | 28±8 | |  |
| Ahmed et al. | 52 | - | - | - | - | - | - | - | | | - | | 123±60 | 28* | |  |
| Lawton et al. | 165 | - | - | - | - | - | - | - | | | - | | 118±46 | 29±8 | |  |
|  |  |  |  |  |  |  |  |  | | |  | |  |  | |  |

Data are presented as raw number or mean and standard deviation. NIRS, noninvasive respiratory support; CRP, C-reactive protein; LDH, lactate dehydrogenase; IL-6, interleukin-6; PaO_2_/FiO_2_, arterial oxygen partial pressure to fractional inspired oxygen ratio; *not available for whole study population.

| **Additional file Table 5.** Pharmacological treatments and awake prone position**.** | | | | | | | | | | |
| --- | --- | --- | --- | --- | --- | --- | --- | --- | --- | --- |
| **Study** | **Patients enrolled with NIRS No.** | | **Hydroxychloroquine No. (%)** | **Antivirals No. (%)** | **Steroids No. (%)** | **Anticoagulation No. (%)** | **Antiplatelets No. (%)** | **Thromboprophylaxis No. (%)** | **Immunomodulators No. (%)** | **Awake prone position (yes/no)** |
|  | |  |  |  |  |  |  |  |  |  |
| Menzella et al. | | 79 | 75 (95) | 41 (52) | 55 (70) | 20 (25) | - | - | 41 (52) | - |
| Franco et al. | | 507 | 135 (27) | 28 (6) | 170 (34) | 61 (12) | - | 27 (5) | 114 (22) | yes |
| Ramirez et al. | | 90 | - | - | - | - | - | - | - | yes |
| Avdeev et al. | | 61 | - | - | - | - | - | - | - | yes |
| Brusasco et al. | | 64 | 64 (100) | - | 64 (100) | - | - | - | - | no |
| Duca et al. | | 78 | - | - | - | - | - | - | - | - |
| Aliberti et al. | | 157 | 152 (97) | 90 (59) | 72 (46) | 45 (29) | - | - | 59 (38) | yes |
| Di Domenico et al. | | 90 | - | - | - | - | - | - | - | - |
| Faraone et al. | | 50 | - | - | 35 (70) | 20 (40) | - | 30 (60) | 17 (24) | - |
| Potalivo et al. | | 71 | 71 (100) | 71 (100) | 57 (80) | - | - | - | 60 (84) | - |
| Di Lecce et al. | | 78 | 71 (91) | 35 (45) | 25 (32) | 78 (100) | - | 18 (23) | - | yes |
| Bellani et al. | | 798 | - | - | - | - | - | - | - | - |
| Vaschetto et al. | | 537 | - | - | - | - | - | - | - | - |
| Coppadoro et al. | | 306 | - | - | - | - | - | - | - | yes |
| Gidaro et al. | | 194 | 120 (62) | 120 (62) | 19 (16) | 20 (10) | 80 (41) | 174 (92) | 65 (34) | yes |
| Ahmed et al. | | 52 | - | - | - | - | - | - | - | - |
| Lawton et al. | | 165 | - | - | - | - | - | - | - | yes |
|  | |  |  |  |  |  |  |  |  |  |

Data are presented as number and percentage in brackets. Also, the application of awake prone position was reported. NIRS, noninvasive respiratory support

**Additional file Table 6.** Noninvasive respiratory support devices and settings.

|  |  | | | | | | |  |
| --- | --- | --- | --- | --- | --- | --- | --- | --- |
| **Study** | | **Patients enrolled with NIRS No.** | **CPAP**  **No.** | **PSV**  **No.** | **Helmet**  **No.** | **Mask**  **No.** | **PEEP (cmH_2_O)** | **FiO_2_**  **(%)** |
|  | |  |  |  |  |  |  |  |
| Menzella et al. | | 79 | - | - | 0 | 79 | - | - |
| Franco et al. | | 507 | 330 | 177 | - | - | 10±2* | - |
| Ramirez et al. | | 90 | - | - | - | - | 10 | 53±45 |
| Avdeev et al. | | 61 | 45 | 16 | 0 | 61 | 10 | - |
| Brusasco et al. | | 64 | 64 | 0 | 39 | 25 | 10 | 60 |
| Duca et al. | | 78 | 71 | 7 | 71 | 7 | 15±4* | 60* |
| Aliberti et al. | | 157 | 157 | 0 | 157 | 0 | 11±2 | 60±7 |
| Di Domenico et al. | | 90 | - | - | - | - | - | - |
| Faraone et al. | | 50 | 25 | 25 | 0 | 50 | - | - |
| Potalivo et al. | | 71 | - | - | 71 | 0 | - | - |
| Di Lecce et al. | | 78 | 40 | 38 | 1 | 77 | 12±2 | 67±18 |
| Bellani et al. | | 798 | 778^§^ | 90^§^ | 617^§^ | 248^§^ | 11±2* | 68±21* |
| Vaschetto et al. | | 537 | 537 | 0 | 399* | 123* | 11±2 | 50 |
| Coppadoro et al. | | 306 | 306 | 0 | 306 | 0 | 7±4 | 63±30 |
| Gidaro et al. | | 194 | 194 | 0 | 194 | 0 | 10±2 | 63±9 |
| Ahmed et al. | | 52 | 52 | 0 | 0 | 52 | - | - |
| Lawton et al. | | 165 | 165 | 0 | 0 | 165 | - | 50 |
|  | |  |  |  |  |  |  |  |

Data are presented as raw number or mean and standard deviation. NIRS, noninvasive respiratory support; CPAP, continuous positive airway pressure; PSV, pressure support ventilation; PEEP, positive end-expiratory pressure; FiO_2_, inspired oxygen fraction. *not available for the whole study population; ^§^data referring to the entire population under NIV at enrollment, not the one in which the outcome could be determined.

| **Additional file Table 7.** Presence of clear criteria and reasons for endotracheal intubation. | | | | | |
| --- | --- | --- | --- | --- | --- |
| **Study** | **ETI Criteria** | **Decreased level of consciousness No.** | **Exhaustion**  **No.** | **Refractory Hypoxemia No.** | **Other**  **No.** |
|  |  |  |  |  |  |
| Menzella et al. | Reported | 0 | 0 | 21 | 0 |
| Franco et al. | Not clearly stated | - | - | - | - |
| Ramirez et al. | Not clearly stated | - | - | - | - |
| Avdeev et al. | Reported | 2 | 3 | 12 | 0 |
| Brusasco et al. | Reported | 0 | 3 | 4 | 0 |
| Duca et al. | Reported | - | - | 26 | - |
| Aliberti et al. | Reported | 0 | 0 | 34 | 0 |
| Di Domenico et al. | Reported | - | - | 36 | - |
| Faraone et al. | Not clearly stated | - | - | - | - |
| Potalivo et al. | Not clearly stated | - | - | - | - |
| Di Lecce et al. | Not reported | - | - | - | - |
| Bellani et al. | Not reported | - | - | - | - |
| Vaschetto et al. | Reported | - | - | - | - |
| Coppadoro et al. | Not Reported | - | 5 | 47 | 2 |
| Gidaro et al. | Not reported | 4 | 3 | 35 | - |
| Ahmed et al. | Not reported | - | - | - | 0 |
| Lawton et al. | Reported | - | - | - | - |
|  |  |  |  |  |  |

Data are presented as raw number; ETI, endotracheal intubation.

| **Additional file table 8.** Criteria for endotracheal intubation (when reported). | | | | | | | |  |
| --- | --- | --- | --- | --- | --- | --- | --- | --- |
| **Menzella et al.** | **Avdeev et al.** | **Brusasco et al.** | **Duca et al.** | **Aliberti et al.** | **Di Domenico et al.** | **Vaschetto et al.** | **Lawton et al.** |  |
|  |  |  |  |  |  |  |  |  |
|  |  |  |  |  |  |  |  |  |
|  |  |  |  |  |  |  |  |  |
| - PaO_2_/FiO_2_ ratio <100 mmHg, RR >36/min (NIV); | - Worsening ARF with respiratory distress; | - 4 days of unsuccessful CPAP; | - PaO_2_ <60 mmHg on NIV with 100% FiO_2_; | - Either 1 major or two minor criteria for >1 h. | - SpO_2_ <90% with oxygen 12 L/min or CPAP/NIV; | - Cardiac or respiratory arrest; | - RR ≥20 and SpO_2_ ≤94% and ≥15 L/min oxygen |  |
| - Coma or convulsive disorder; | - SpO_2_ below 88% without response to NIV; | - PaO_2_/FIO_2_ tending to decrease; | - Age, comorbidities and ARF severity. | - Major criteria | - PaO_2_/FiO_2_ <200 mmHg; | - Inability to protect the airway; | - PaO_2_/FiO_2_ <200mmHg |  |
| - Abundant tracheal and/or bronchial secretions; | - Respiratory acidosis with a pH below 7.30; | - RR >30/min; |  | - Respiratory arrest; | - Persistent respiratory fatigue. | - Coma or psychomotor agitation; |  |  |
| - Hemodynamic or ECG instability. | - Hemodynamic instability; | - PaO_2_ <60 mmHg. |  | - Respiratory pause with unconsciousness; |  | - Unmanageable secretions or uncontrolled vomiting; |  |  |
|  | - Exhaustion. |  |  | - Severe haemodynamic instability; |  | - Life-threatening arrhythmias or ECG signs of ischemia; |  |  |
|  |  |  |  | - Intolerance to helmet |  | - Hemodynamic instability; |  |  |
|  |  |  |  | - Minor criteria |  | - Intolerance to all interfaces; |  |  |
|  |  |  |  | - Reduction of ⩾30% of basal PaO_2_/FIO_2_ ratio; |  | - Dyspnoea or RR >30 breaths/min during CPAP; |  |  |
|  |  |  |  | - PaO_2_/FIO_2_ ratio <100; |  | - SpO_2_ <92% or pH < 7.35 during CPAP. |  |  |
|  |  |  |  | - 20% increase of arterial carbon dioxide tension; |  |  |  |  |
|  |  |  |  | - Worsening of alertness; |  |  |  |  |
|  |  |  |  | - New onset or persistent respiratory distress; |  |  |  |  |
|  |  |  |  | - SpO_2_ <90%; |  |  |  |  |
|  |  |  |  | - Exhaustion. |  |  |  |  |

PaO_2_/FiO_2_, arterial oxygen partial pressure to fractional inspired oxygen ratio; RR, respiratory rate; NIV, non-invasive ventilation, ECG, electrocardiography; ARF, acute respiratory failure, SpO_2_, peripheral oxygen saturation; CPAP, continuous positive airway pressure.

| **Additional file table 9.** Hospital length of stay, noninvasive ventilation and mechanical ventilation duration, time-lag between noninvasive ventilation and intubation. |
| --- |

| **Study** | **Patients enrolled with NIV No.** | | **Hospital LOS**  **(days)** | **NIV duration (days)** | **IMV duration**  **(days)** | **Total duration NIV+IMV (days)** | **NIV-to-ETI time-lag (hours)** |
| --- | --- | --- | --- | --- | --- | --- | --- |
|  | |  |  |  |  |  |  |
| Menzella et al. | | 79 | - | - | - | - | - |
| Franco et al. | | 507 | 20±13 | - | - | - | - |
| Ramirez et al. | | 90 | - | 12±8 | - | - | - |
| Avdeev et al. | | 61 | - | - | - | - | - |
| Brusasco et al. | | 64 | 19 | 8±4 | 11 | 15 | 81±32 |
| Duca et al. | | 78 | - | - | - | - | - |
| Aliberti et al. | | 157 | - | - | - | - | 72±53 |
| Di Domenico et al. | | 90 | - | - | - | - | - |
| Faraone et al. | | 50 | 20±15 | 9±8 | - | - | 55±81 |
| Potalivo et al. | | 71 | 26±27 | 4±2 | 20 | 23 | 85 |
| Di Lecce et al. | | 78 | 17±11 | - | - | - | 36±33 |
| Bellani et al. | | 798 | - | - | - | - | 137 |
| Vaschetto et al. | | 537 | 17±13 | 4±5 | - | - | - |
| Coppadoro et al. | | 306 | - | 6 | - | - | 113±72 |
| Gidaro et al. | | 194 | 21±15 | 7±6 | - | - | - |
| Ahmed et al. | | 52 | - | 2 | - | - | - |
| Lawton et al. | | 165 | - | - | - | - | - |
|  | |  |  |  |  |  |  |

Data are presented as raw number or mean and standard deviation (when available). LOS, length of stay; NIV, non-invasive ventilation; IMV, invasive mechanical ventilation; ETI, endotracheal intubation.

| **Additional table 10.** Peer-reviewed studies excluded after full-text reading | | |
| --- | --- | --- |
| Title | Author | Reason for exclusion |
| Clinical outcomes in diabetic vs non-diabetic patients with severe covid-19 | Poon et al. | < 50 patients; no data about type of NIRS and related outcome |
| The association of race, ethnicity, and outcomes of patients with covid-19 in new york city | Maeda et al. | Outcome |
| A nomogram to predict the risk of unfavourable outcome in COVID-19: a retrospective cohort of 279 hospitalized patients in Paris area | Nguyen et al. | <50 patients |
| Patient characteristics and predictors of mortality in 470 adults admitted to a district general hospital in England with Covid-19. | Thompson et al. | <50 patients |
| Effect of tocilizumab in hospitalized patients with severe COVID-19 pneumonia: A case-control cohort study | Rossi et al. | <50 patients |
| Clinical characteristics and outcomes of critically ill patients with novel coronavirus infectious disease (COVID-19) in China: a retrospective multicenter study | Xie et al. | NIRS in ICU |
| Clinical Characteristics and Outcomes of Non-ICU Hospitalization for COVID-19 in a Nonepicenter, Centrally Monitored Healthcare System | Nemer et al. | <50 patients |
| Incidence of deep vein thrombosis among non-ICU patients hospitalized for COVID-19 despite pharmacological thromboprophylaxis | Santoliquido et al. | <50 patients |
| Outcomes of COVID-19 hospitalized patients previously treated with renin-angiotensin system inhibitors | Cordeanu et al | <50 patients, no data about type of NIRS, location of NIRS and related outcome |
| Baseline Characteristics and Outcomes of 1591 Patients Infected with SARS-CoV-2 Admitted to ICUs of the Lombardy Region, Italy | Grasselli et al | NIRS in ICU |
| Clinical characteristics and laboratory indicator analysis of 67 COVID-19 pneumonia patients in Suzhou, China | Wang et al | <50 patients |
| Continuous positive airway pressure to avoid intubation in SARS-CoV-2 pneumonia: a two-period retrospective case-control study. | Oranger et al. | < 50 patients, no agreement for data sharing |
| Clinical characteristics and day-90 outcomes of 4244 critically ill adults with COVID-19: a prospective cohort study. | Scmidth et al | NIRS in ICU |
| Beneficial effect of corticosteroids in preventing mortality in patients receiving tocilizumab to treat severe COVID-19 illness | Rubio-Rivas et al | No data of NIRS outcome |
| ﻿CPAP management of COVID-19 respiratory failure: a first quantitative analysis from an inpatient service evaluation. | Ashish et al | <50 patients |
| Characteristics and outcomes of patients with COVID-19 at a district general hospital in Surrey, UK. | Knights et al | <50 patients |
| Continuous Positive Airway Pressure (CPAP) face-mask ventilation is an easy and cheap option to manage a massive influx of patients presenting acute respiratory failure during the SARS-CoV-2 outbreak: A retrospective cohort study | Alviset et al | <50 patients |
| Coronavirus disease 2019 (COVID-19) associated coagulopathy and its impact on outcomes in Shenzhen, China: A retrospective cohort study | Luan et al | <50 patients |
| Characteristics of mechanically ventilated patients with COVID-19 and persons under investigation negative for COVID-19 at an academic medical center: A retrospective cross-sectional pilot study. | Lam et al | <50 patients |
| Clinical characteristics, symptoms and outcomes of 1054 adults presenting to hospital with suspected COVID-19: a comparison of patients with and without SARS-CoV-2 infection | Brendish et al | No data about type of NIRS, location of NIRS and related outcome |
| Clinical characteristics and outcomes of immunosuppressed patients hospitalized with COVID-19: experience from London. | Vaid et al | No data about location of NIRS and related outcome. Composite outcome |
| Helmet Continuous Positive Airway Pressure in the Treatment of COVID-19 Patients with Acute Respiratory Failure could be an Effective Strategy: A Feasibility Study. | Alharthy et al | <50 patients |
| Effect of Hydroxychloroquine on Clinical Status at 14 Days in Hospitalized Patients with COVID-19: A Randomized Clinical Trial | Self et al | No data about type of NIRS, location of NIRS and related outcome |
| An integrated multidisciplinary model of COVID-19 recovery care. | O'brien et al | No data about type of NIRS, < 50 patients |
| Risk factors for non-invasive/invasive ventilatory support in patients with COVID-19 pneumonia: A retrospective study within a multidisciplinary approach | Suardi et al | <50 patients |
| ﻿At the peak of Covid-19 age and disease severity but not comorbidities are predictors of mortality. Covid-19 burden in Bergamo, Italy. | Novelli et al | Same group of Duca, no data about location of NIRS |
| Preparedness and response to the covid-19 emergency: Experience from the teaching hospital of Pisa, italy | Baggiani et al | Main outcome |
| Prone positioning in patients treated with non-invasive ventilation for COVID-19 pneumonia in an Italian emergency department. | Bastoni et al | <50 patients |
| Continuous positive airway pressure-treated patients' behaviors during the COVID-19 crisis. | Pepin et al | Main outcome |
| Corticosteroids for COVID-19 patients requiring oxygen support? Yes, but not for everyone: Effect of corticosteroids on mortality and intensive care unit admission in patients with COVID-19 according to patients' oxygen requirements. | Tortajada et al | No data about type of NIRS, location of NIRS and related outcome |
| Effects of Comorbid Factors on Prognosis of Three Different Geriatric Groups with COVID-19 Diagnosis | Görgülü et al | <50 patients |
| Effect of Tocilizumab vs Usual Care in Adults Hospitalized With COVID-19 and Moderate or Severe Pneumonia: A Randomized Clinical Trial. | Hermine et al | <50 patients |
| Characteristics, comorbidities and survival analysis of young adults hospitalized with COVID-19 in New York City. | Altonen et al | <50 patients |
| Epidemiology, outcomes, and the use of intensive care unit resources of critically ill patients diagnosed with COVID-19 in Sao Paulo, Brazil: A cohort study. | Socolovithc | Outocme, NIRS location |
| Feasibility and physiological effects of prone positioning in non-intubated patients with acute respiratory failure due to COVID-19 (PRON-COVID): a prospective cohort study | Coppo et al | Same group of Coppadoro |
| Epidemiology, outcomes, and the use of intensive care unit resources of critically ill patients diagnosed with COVID-19 in Sao Paulo, Brazil: a cohort study | Socolovitch et al | NIRS location |
| Eculizumab treatment in patients with COVID-19: preliminary results from real life ASL Napoli 2 Nord experience. | Diurno et al | <50 patients |
| Clinical characteristics and predictors of survival in adults with coronavirus disease 2019 receiving tocilizumab | Morrison et al | Outcome, NIRS location, < 50 patients |
| Clinical characteristics of coronavirus disease 2019 in China | Guan et al | No data about NIRS location and type of NIRS with related outcome |
| Prone Positioning for Pregnant Women With Hypoxemia Due to Coronavirus Disease 2019 (COVID-19). | Tolcher et al | <50 patients |
| A comprehensive strategy for the early treatment of COVID-19 with azithromycin/hydroxychloroquine and/or corticosteroids: Results of a retrospective observational study in the French overseas department of Réunion Island. | Dubernet et al. | <50 patients |
| Outcomes of COVID-19 in 79 patients with IBD in Italy: an IG-IBD study. | Bezzio et al | <50 patients |
| Outcome of non-invasive ventilation in COVID-19 critically ill patients: A Retrospective observational Study. | Mukthar et al | NIRS location, < 50 patients |
| Severity of respiratory failure at admission and in-hospital mortality in patients with COVID-19: a prospective observational multicentre study. | Santus et al | <50 patients in the emergency dept, no data of CPAP location in hospital and related outcome |
| COVID-19-associated hyperinflammation and escalation of patient care: a retrospective longitudinal cohort study. | Manson et al | No data about NIRS location |
| Non-severe immunosuppression might be associated with a lower risk of moderate-severe acute respiratory distress syndrome in COVID-19: A pilot study. | Monreal et al | No data about NIRS location and type of NIRS |
| Case characteristics, resource use, and outcomes of 10 021 patients with COVID-19 admitted to 920 German hospitals: an observational study. | Karagiannidis et al | No data about NIRS location and type of NIRS with related outcome |
| Clinical characteristics, management and in-hospital mortality of patients with coronavirus disease 2019 in Genoa, Italy | Vena et al | No data about NIRS location and type of NIRS with related outcome |
| Efficacy and safety of tocilizumab in severe COVID-19 patients: a single-center retrospective cohort study. | Campochiaro et al | <50 patients, no data about NIRS location and type of NIRS with related outcome |
| Bilevel and continuous positive airway pressure and factors linked to all-cause mortality in COVID-19 patients in an intermediate respiratory intensive care unit in Italy. | Carpagnano et al | Same group of Di Lecce |
| Tocilizumab for the treatment of severe COVID-19 pneumonia with hyperinflammatory syndrome and acute respiratory failure: A single center study of 100 patients in Brescia, Italy | Toniati et al | No data about NIRS location and type of NIRS with related outcome |
| Use of critical care resources during the first 2 weeks (February 24–March 8, 2020) of the Covid-19 outbreak in Italy | Tonetti et al | Incomplete data about NIRS outcome after discussing with corresponding author and principal investigation |
| Prone and Lateral Positioning in Spontaneously Breathing Patients With COVID-19 Pneumonia Undergoing Noninvasive Helmet CPAP Treatment | Retucci et al | < 50 patients |
| Respiratory Parameters in Patients With COVID-19 After Using Noninvasive Ventilation in the Prone Position Outside the Intensive Care Unit | Sartini et al | < 50 patients |

NIRS, noninvasive respiratory support, ICU, intensive care unit

| **Additional table 11.** Pre-prints excluded after full-text reading | | |
| --- | --- | --- |
| Title | Author | Reason for exclusion |
| Clinical Course And Risk Factors For In-hospital Death In Critical COVID-19 In Wuhan, China | Li et al. | NIRS in ICU |
| The Presence of Ambulatory Hypoxia as an Early Predictor of Moderate to Severe COVID-19 Disease | Bhasin et al. | NIRS in ICU |
| Azithromycin in Hospitalised Patients with COVID-19 (RECOVERY): a randomised, controlled, open-label, platform trial | Horby et al. | NIRS in ICU |
| Cardiovascular disease and severe hypoxemia associated with higher rates of non-invasive respiratory support failure in COVID-19 | Wang et al. | NIRS in ICU |
| SARS-COV-2 comorbidity network and outcome in hospitalized patients in Crema, Italy | Benelli et al. | No clear data on NIRS location |
| Clinical characteristics of critically ill patients with COVID-19 | Carboni Bisso et al. | NIRS in ICU |
| Combination therapy with tocilizumab and corticosteroids for aged patients with severe COVID-19 pneumonia: a single-center retrospective study. | Lopez-Medrano et al. | No clear data on NIRS location |
| Characteristics of patients with COVID-19 during epidemic ongoing outbreak in Wuhan, China | Luo et al. | < 50 patients |
| Descriptive epidemiology of 16,780 hospitalized COVID-19 patients in the United States | Rizzo et al. | NIRS location |
| Tocilizumab in Hospitalized Patients With COVID-19 Pneumonia | Rosas et al. | Composite outcome |
| Hydroxychloroquine with or without azithromycin and in-hospital mortality or discharge in patients hospitalized for COVID-19 infection: a cohort study of 4,642 in-patients in France | Sbidian et al. | NIRS in ICU |
| Anakinra and Intravenous IgG versus Tocilizumab in the Treatment of COVID-19 Pneumonia | Zantah et al. | No clear data on NIRS location |
| Clinical features and outcomes of 221 patients with COVID-19 in Wuhan, China | Zhang et al. | NIRS in ICU |
| Role of intermediate care unit admission and non-invasive respiratory support during the COVID-19 pandemic: a retrospective cohort study | Grosgurin et al. | No clear data of NIRS type and related outcome. Emailing attempt without no reply |
| Incidence and Characteristics of Co-infection and Secondary Infection in Patients with COVID-19 | Guo et al. | <50 patients |
| Randomized controlled trial of convalescent plasma therapy against standard therapy in patients with severe COVID-19 disease | AlQahtani et al. | <50 patients |
| Continuous positive airway pressure face-mask ventilation to manage massive influx of patients requiring respiratory support during the SARS-CoV-2 outbreak | Alviset et al. | <50 patients |
| Early CPAP reduced mortality in covid-19 patients. Audit results from Wrightington, Wigan and Leigh Teaching Hospitals NHS Foundation Trust | Ashish et al. | <50 patients |
| Effectiveness of Convalescent Plasma for Treatment of COVID-19 Patients | Chen et al. | <50 patients |
| Favipiravir versus Arbidol for COVID-19: A Randomized Clinical Trial | Chen et al. | <50 patients |
| Efficacy and safety of interferon β-1a in treatment of severe COVID-19: A randomized clinical trial | Davoudi-Monfared et al. | <50 patients |
| Acute Lung injury evolution in Covid-19 | Doglioni et al. | <50 patients |
| Early Awake Prone and Lateral Position in Non-intubated Severe and Critical Patients with COVID-19 in Wuhan: A Respective Cohort Study | Dong et al. | <50 patients |
| 68 Consecutive patients assessed for COVID-19 infection; experience from a UK regional infectious disease unit | Easom et al. | <50 patients |
| Effectiveness of remdesivir with and without dexamethasone in hospitalized patients with COVID-19 | Garibaldi et al. | <50 patients |
| Association of Obstructive Sleep Apnea and severity of COVID-19: A hospital based observational study | Kar et al. | <50 patients |
| Laboratory biomarkers associated with COVID-19 severity and management. | Keddie et al. | <50 patients |
| A two-arm, randomized, controlled, multi-centric, open-label Phase-2 study to evaluate the efficacy and safety of Itolizumab in moderate to severe ARDS patients due to COVID-19 | Kumar et al. | <50 patients |
| Gastrointestinal involvement attenuates COVID-19 severity and mortality | Livanos et al. | <50 patients |
| COVID-19 Case Series at UnityPoint Health St. Lukes Hospital in Cedar Rapids, IA | McGrail et al. | <50 patients |
| No associations between physical activity and clinical outcomes among hospitalized patients with severe COVID-19 | Pinto et al. | <50 patients |
| Effect of tocilizumab in hospitalized patients with severe pneumonia COVID-19: a cohort study | Rossi et al. | <50 patients |
| Feasibility of non-invasive nitric oxide inhalation in acute hypoxemic respiratory failure: potential role during the COVID-19 pandemic | Shekar et al. | <50 patients |

NIRS, non-invasive respiratory; ICU, intensive care unit
